# Effects of tris (hydroxymethyl) aminomethane and egg yolk on the cryopreservation of buck semen

**DOI:** 10.5455/javar.2022.i636

**Published:** 2022-12-31

**Authors:** Md. Mostofa Kamal, Md. Emtiaj Alam, Md. Akhtarul Islam, Md. Royhan Gofur, Aurangazeb Kabir

**Affiliations:** Department of Veterinary and Animal Sciences, Faculty of Veterinary and Animal Sciences, Rajshahi University, Rajshahi, Bangladesh

**Keywords:** Sperm, tris, egg yolk, freezing, thawing, semen quality

## Abstract

**Objectives:**

This study was designed to examine the effects of various concentrations of tris (hydroxymethyl) aminomethane (tris) and egg yolk on the quality of cryopreserved buck sperm.

**Materials and Methods:**

The collected semen samples were pooled, washed, and diluted into five different freezing extender groups, *viz*., extender I (tris 0% + egg yolk 0%), extender II (tris 1.41% + egg yolk 4%), extender III (tris 2.41% + egg yolk 8%), extender IV (tris 3.41% + egg yolk 16%), and extender V (tris 4.41% + egg yolk 24%). The sperm parameter of the five groups of extenders was evaluated after equilibration and cryopreservation.

**Results:**

The results showed that extenders II–V provided significantly higher semen progressive motility and total motility percentages than extender I after equilibration (*p* < 0.05). The higher percentages of semen progressive motility, total motility, viability, and plasma membrane integrity (by both HOST under light microscopy and stain after HOST under light microscopy) were found in the sperm cryopreserved with extender IV than extender I, extender II, and extender III groups after thawing (*p* < 0.05). In addition, semen progressive motility, total motility, and viability were not further increased, or plasma membrane integrity (by both HOST tests) was decreased by the addition of tris and egg yolk (extender V) after cryopreservation (*p* < 0.05).

**Conclusion:**

In conclusion, our result indicates that the following washing, the supplementation of tris (3.41% + egg yolk 16%) on the freezing extender are suitable for improving the semen quality of buck after freezing and thawing.

## Introduction

Cryopreservation is an effective technique that helps to preserve the structure and function of semen. It also aids in the advancement of the goat breeding program. However, during cryopreservation, the spermatozoa face different ranges of temperatures, leading them to experience cryoinjuries of different types. The functional maintenance of semen is reduced due to intracellular ice crystal formation, oxidative stress, osmotic pressure, pH, and cold shock during the freezing and thawing processes [1–5]. So, the semen extender for cryopreservation should have energy sources like fructose, glucose, and trehalose; cryoprotectants like glycerol; a well-buffered agent (tris) to keep the pH level stable; and a cold shock protector like egg yolk. The pH of freshly collected buck semen was controlled at around 7.02 ± 0.19 [6,7]. However, the sperm pH was decreased during the glycolytic pathway for energy production, which could reduce the metabolism and motility of sperm [8]. So, it is crucial to control semen pH throughout the semen cryopreservation steps. Tris is used in semen extenders as a pH regulator [9].

In addition, the egg yolk extender controls cholesterol efflux, integral protein, and phospholipids and defends the plasma membrane as opposed to temperature-related damage [10]. However, the previous study reported that the egg yolk acts with the glycoproteins-60 (BUSgp60), which is secreted from the bulbourethral gland. The BUSgp60 has triacylglycerol hydrolase activity, which disrupts the cell membrane, resulting in a decrease in spermatozoa motility [11]. In addition, egg yolk phosphatidylcholine can be transformed into fatty acids and lysophosphatidylcholine (LPC) by phospholipase A, where LPC harms the biomembrane [12]. As a result, these reactions influence cryocapacitation, the acrosome defect, loss of motility, and the viability of the semen [12,13]. The protocol has been proposed to decrease these interactive losses, including washing semen [14] and using an appropriate level of egg yolk [15–17] on semen extenders.

However, washing sperm removes the antioxidative enzymes and protective enzymes, whereas the egg yolk concentration influences the cryoprotective ability of the semen extender [18]. So it indicates that the various extrinsic and intrinsic factors regulate the standard of sperm during different stages of cryopreservation.

The various concentrations of tris and egg yolk were added separately to the frozen semen extenders of different animals in the different studies and affected semen quality after freezing and thawing [18–20]. However, the synergistic effects of using different combined concentrations of tris and egg yolk supplementation in the freezing extender for buck spermatozoa have not been well explained. In this study, we examined the synergistic effect of tris and egg yolk concentration on the freezing extenders for the quality of the freeze-thaw goat spermatozoa.

## Materials and Methods

### Ethical statement

The experiment was approved by the Institutional Animal, Medical Ethics, Biosafety and Biosecurity Committee (IAMEBBC) of Rajshahi University, Bangladesh.

### Animal selection

Three sexually mature beetal bucks were selected for this study. They were between the 15th and 30th months of age and weighed over 50 kg, and the body condition score was 2.70 (scale: 1–5, where 1 = was emaciated. and 5 = grossly over-condition). All bucks were kept and maintained at the veterinary clinics and artificial insemination center, Naricalbaria, under the Faculty of Veterinary and Animal Sciences of the University of Rajshahi. The experimental bucks were kept in buck sheds with natural ambiance and were given 600 gm of concentrate feed, 400 gm rice straw, and 2 kg of Napier grass, along with an ad libitum amount of fresh water each day/buck. 

### Semen collection

Following stimulation with a doe, we collected the semen twice a week (≥5-day intervals) using an artificial vagina. A total of 12 aliquots (4/goat) were used in this study. Soon after collection, the tube containing sperm was kept in a water bath at 37°C. The sperm parameters like volume (ml), color, mass movement (0–4), semen concentration (*n* × 10^9^), sperm progressive motility (SPM), sperm total motility (STM), sperm viability (SV), sperm plasma membrane integrity (SPMI) by hypoosmotic swelling test (HOST) under bright field without stain and bright field after HOST following stain were evaluated. All sperm parameters were assessed visually by the same two researchers, who worked independently throughout the study to improve the accuracy of the result. Ejaculates with a volume ≥0.65 ml, color creamy white, mass movement ≥3.20, concentration ≥1.75 × 10^9^ sperm/ml, STM ≥80%, SV ≥80%, and SPMI ≥70% were selected for this study.

### Semen extenders

The extender without tris and egg yolk (extender I) is considered a control which is composed of citric acid (Sigma-Aldrich Corp., St. Louis, MO 63103, U.S.A), 1.51 gm, fructose (Himedia Lab. Pvt., Ltd, Mumbai 400086, India) 0.81 gm, gentamycin (Opsonin Pharma, Ltd., Barishal, Bangladesh) 250 µg/ml, glycerol (Riedel-de Haen TM, D-30926 Seelze, Germany) 3.5% and deionized water up to 100 ml, pH were adjusted to 7.2. Additionally, extender II, extender III, extender IV, and extender V were made by using tris (Himedia Lab. Pvt.,) 1.41 gm + egg yolk 4 ml, tris 2.41 gm + egg yolk 8 ml, tris 3.41 gm + egg yolk 16 ml, tris 4.41 gm + egg yolk 24 ml, respectively, where the other ingredients were same like control (Table 1). Each extender is divided into Parts 1 (without glycerol) and 2 (with 7% glycerol, for a final concentration of 3.5% glycerol).

### Sperm progressive motility and total motility

SPM and STM were checked after 10 µl sperm were mixed with prewarmed (at 37°C) washing media (tris 2.41 gm, citric acid 1.51 gm, fructose 0.81 gm, gentamicin 250 µg/ml, deionized water until 100 ml, pH 7.2). An aliquot of 10 µl of sperm was taken on the prewarmed, clean glass slide, then overlaid with a prewarmed coverslip. The bright-field microscope at 400× was used to analyze 200 sperm from at least three separate fields. Progressive and non-progressive motility were counted according to the guidelines of WHO [21].

### Sperm viability

The eosin-nigrosin staining procedure was used to determine the viability of the sperm. In brief, 3% trisodium citrate dihydrate (Himedia Lab. Pvt., Ltd.), 1% eosin (Sigma-Aldrich Corp.), and 5% nigrosin (Himedia Lab. Pvt., Ltd.) were dissolved in distilled water. The solution was dissolved by gentle heating and continuous stirring but not by boiling. After dissolving, the solution was cooled down and kept in a 15-ml Falcon tube with aluminum foil in a dark place at room temperature. The 20 µl of sperm and 20 µl of this stain were correctly mixed in an Eppendorf tube and allowed to wait for at least 30 sec. An aliquot of 10 µl of stained semen was transferred on a slide, then smeared and covered by a coverslip, and waited for semen to settle down before evaluation. Two hundred sperm from at least three different fields were checked 400 times under a bright-field microscope, and the percentages of viable (unstained, white heads) and dead (pink/redheads) sperm were counted.

**Table 1.** Composition of the extenders used for the cryopreservation of the goat semen.

Ingredients	Extender I	Extender II	Extender III	Extender IV	Extender V
Tris (gm)	0	1.41	2.41	3.41	4.41
Egg yolk (ml)	0	4	8	16	24
Citric acid (gm)	1.51	1.51	1.51	1.51	1.51
Fructose (gm)	0.81	0.81	0.81	0.81	0.81
Gentamycin (µg/ml)	250	250	250	250	250
Glycerol (ml)	3.5	3.5	3.5	3.5	3.5
Deionized water (ml)	Up to 100	Up to 100	Up to 100	Up to 100	Up to 100
pH	7.2	7.2	7.2	7.2	7.2

### Sperm plasma membrane integrity under light microscopy

The functional integrity of the semen plasma membrane was checked by HOST. This test was performed by Jeyendran et al. [22] and Revell and Mrode [23] with some modifications. In brief, the 0.9 gm fructose (Himedia Lab. Pvt., Ltd.) and 0.49 gm trisodium citrate dihydrate (Himedia Lab. Pvt. Ltd.) dissolved up to 100 ml distilled water. An aliquot of 62.5 µl of sperm was added to 250 µl of prewarmed HOST solution, after which it was incubated at 37°C for 1 h. A 10 µl droplet was kept on a clean glass slide and coated with a prewarmed coverslip. The percentage of spermatozoa showing coiling and swelling of the tail was counted 400 times under a light microscope. At least 3 distinct fields, consisting of 200 sperm, were evaluated. 

### Sperm plasma membrane integrity by HOST following stained under light microscopy

We also examined the functional integrity of sperm by HOST followed by staining according to Baiee et al. [24] with some modifications. In brief, after 1 h of incubation of 62.5 µl sperm with 250 µl HOST solution at 37°C, an aliquot of 10 µl of semen, and 10 µl stain (1% eosin, 5% nigrosin, and 3% trisodium citrate dihydrate in distilled water) were taken on the prewarmed slide and waited for at least 30 sec. After that, they were placed under a coverslip that had been warmed and under a 400× light microscope, the percentage of sperm exhibiting coiling and swelling of the tail was measured. Two hundred sperm from at least three different fields were studied.

### Experimental design

This study was carried out to evaluate the various extenders for quality goat sperm that contained no or different levels of tris and egg yolk. The pooled sample was washed twice in the following ways. The pooled ejaculates were washed with tris-based washing media and centrifuged (at 1,000 rpm) for 5 min. The seminal plasma was washed by aspirating the supernatant. Then add tris-based washing media, mixed adequately by gentle pipetting, separating five equal parts into five Falcon tubes. The tubes were marked with an extender I, extender II, extender III, extender IV, and extender V. Then, each tube was filled with a washing medium and centrifuged for 5 min at 1,000 rpm to wash the semen. Then each tube’s semen plasma was removed by aspirating the supernatant. Each marked tube of semen was mixed with the same group of the first part of the extender without glycerol and stored at 4°C for 2 h. Then the following two experiments were carried out.

The purpose of experiment 1 was to check the effects of five different groups of extenders on spermatozoa quality after equilibration. After gentle pipetting, an aliquot of 10 µl of sperm was added to the prewarmed tris-based washing media and kept at 37°C in the water bath for 5 min. Progressive motility and total motility percentage were evaluated for all five extender groups.

Experiment 2 was conducted to check the effects of without and with various concentrations of tris and egg yolk-containing extenders on semen characteristics after cryopreserved-thawed steps. After equilibration, the second part of each extender, containing 7% glycerol, was added to the solution to obtain the final concentration of 9 × 10^7^ spermatozoa/ml with a final glycerol concentration of 3.5%. Then diluted semen samples were taken into 0.25 ml straws (imv technologies, France). The straws were sealed, then placed horizontally over 4 cm of liquid nitrogen for 12 min before being dropped into the liquid nitrogen storage tank. After >1 day of storage, the straws containing cryopreserved sperm were thawed in a water bath (at 37°C) for 30 sec. The average SPM, STM, SV, and SPMI were evaluated for all different extender groups.

### Statistical analysis

The results were presented as means ± SD. Statistical differences in semen parameters were evaluated via an ANOVA and LSD test (SPSS version 26.0, a statistical package, SPSS Inc., Chicago, IL). *p *< 0.05 was regarded as statistically significant in each case.

## Results

### Effect of extenders for progressive motility and total motility of buck semen after equilibration

Table 2 contains the findings of Experiment 1. The progressive motility and total motility were significantly lower (*p *< 0.05) on extender I than on extenders II, III, IV, and V. The highest percentage of progressive and total motility was found on extender IV. However, the extenders III, IV, and V results did not differ significantly. At the same time, the progressive and total motility was significantly higher (*p *< 0.05) on extenders III, IV, and V than on extender II.

**Table 2.** Seminal parameter status of the bucks after being equilibrated without and with various combinations of tris and egg yolk in five groups of extenders.

Extender	Characteristics of spermatozoa (%)	
	Progressive motility	Total motility
Extender I	3.31 ± 1.95^c^	10.55 ± 5.94^c^
Extender II	48.52 ± 5.82^b^	61.34 ± 7.08^b^
Extender III	70.08 ± 10.02^a^	76.37 ± 9.58^a^
Extender IV	72.08 ± 9.23^a^	81.63 ± 8.38^a^
Extender V	71.20 ± 11.55^a^	79.84 ± 9.72^a^

Values are mean ± SD.

^a–c^different superscripts indicate statistical differences (*p* < 0.05) within a column.

### Effect of extenders for progressive motility, total motility, viability, and plasma membrane integrity of buck semen after cryopreservation

Table 3 shows the effect of extenders on sperm progressive motility, total motility, viability, plasma membrane integrity by HOST (without stain) positive, and plasma membrane integrity by HOST (following stain) positive after the frozen-thawed step.

The proportion of the progressive motility after the frozen-thawed step was significantly higher (*p* < 0.05) in the extenders IV and V groups than in the extenders I, II, and III groups. However, the extenders between IV and V, or I and II, groups showed no significant differences. The progressive motility of extender III was higher than that of the extender I and II groups (*p* < 0.05).

The proportion of the total motility was significantly higher (*p* < 0.05) in the extenders IV and V groups than in the other three groups (extenders I, II, and III). No differences were found between extenders II and III groups; however, extender II and III provide a much more significant percentage (*p* < 0.05) of overall motility than extender I.

The viability of sperm on extenders IV and V was substantially higher (*p* < 0.05) than on extenders I, II, and III. However, the extenders IV and V groups did not significantly differ. The extender II and III groups produce considerably more viable sperm (*p* < 0.05) than the extender I group. Additionally, extender III had a higher SV than extender II (*p* < 0.05).

In both tests for plasma membrane integrity, HOST without stain and HOST following stain under light microscopy, the highest percentages of plasma membrane integrity were observed on extender IV, and the result was significantly higher (*p* < 0.05) than the extenders I, II, III, and V groups. 

**Table 3.** Seminal parameter status of the bucks cryopreserved without and with various combinations of tris and egg yolk in five groups of extenders.

Extender		Characteristics of spermatozoa (%)			
	Progressive motility	Total motility	Viability	HOST positive without stain	HOST positive following stained
Extender I	1.10 ± 1.35^c^	3.86 ± 3.48^c^	17.24 ± 4.36^d^	14.66 ± 2.92^d^	15.24 ± 1.97^e^
Extender II	2.40 ± 0.98^c^	13.47 ± 2.38^b^	26.71 ± 5.39^c^	17.09 ± 1.65^d^	21.67 ± 1.93^d^
Extender III	10.29 ± 2.14^b^	17.78 ± 2.43^b^	35.05 ± 6.67^b^	21.61 ± 1.43^c^	27.08 ± 1.78^c^
Extender IV	27.34 ± 7.47^a^	42.92 ± 4.12^a^	51.66 ± 5.79^a^	31.12 ± 4.80^a^	38.80 ± 2.02^a^
Extender V	32.12 ± 5.10^a^	40.01 ± 5.22^a^	48.83 ± 2.66^a^	25.77 ± 2.70^b^	33.52 ± 3.55^b^

Values are mean ± SD.

^a–e^different superscripts indicate statistical differences (*p* < 0.05) within a column.

## Discussion

During freezing, the sperm are exposed to a wide range of temperatures, which causes structural and functional changes. So, for long-term sperm preservation until artificial insemination, the spermatozoa need an extender containing appropriate cryoprotectants, a sufficient quantity of nutrients, a well-buffered agent like tris, and a cold shock protector like egg yolk during the storage in liquid nitrogen [9,25–31]. Moreover, cryopreservation still causes semen damage in various animals [32,33], and researchers are still determining the optimum ingredient levels of the semen extender for freezing buck semen.

The freshly collected buck semen pH is tightly regulated and controlled at around 7.02 [6,7]. So it is necessary to maintain this pH in the extender to maintain a well-buffering system to support semen survival. During semen cryopreservation, any changes in pH could affect the metabolism rates, motility, viability, and plasma membrane integrity [34,35]. Sodium citrate, HEPES, bicarbonate, and tris have been used as pH controllers to maintain the pH in the extender [9,19,25]. The pH of tris is representative of most living cells. It has the advantage of being much less expensive than any other buffering agent and being highly soluble in water. In mammals, tris has been applied in the semen extender as a practical pH regulator that prevents the alteration of the hydroxide ion and hydronium ion concentration, protecting the cell against pH changes [19].

The egg yolk is a crucial component of the semen extender, which serves as a non-permeable cryoprotectant and inhibits plasma membrane degradation. It contains low-density lipoprotein, cholesterol, and phospholipids, which protect the functional integrity of the semen against cold shock and cryoinjuries [36–40]. As well, egg yolk protects sperm during cryopreservation; simultaneously, it reacts with phospholipase A2 and glycoprotein 60 of seminal plasma, decreasing the motility and causing the death of sperm [11,41].

In experiment 1, the effects of various extenders on progressive and total motility following semen equilibration are shown in Table 2. Progressive and total motility was significantly decreased in the extenders I compared with the II, III, IV, and V groups. Semen washing is suspected to be helpful in bucks as it removes the reactive protein of seminal plasma, but the process concurrently eliminates the antioxidative enzymes and the protective proteins [13]. The earlier report also showed that the semen is exposed to a lower temperature during equilibration, which influences the efflux of membrane constituents like cholesterol. Reactive oxygen species (ROS) production also increased [42–44]. The plasma membrane and acrosome are damaged by cryoinjury due to the loss of integral proteins and cholesterol [45]. The presence of tris and egg yolk in the semen extender decreased the loss of plasma membrane permeability, integrity, and viability [46,47] during cool storage. So, this study proved the exclusion of seminal plasma before freezing buck sperm and stored the progressive motility and total motility by using tris and egg yolk [48–50] because extender I did not contain any tris or egg yolk; however, extender II to extender V contains tris and egg yolk. 

 In the second experiment, we found that extender IV, containing 3.41% tris and 16% egg yolk provided a significantly best result after freezing-thawing than extender I (0% tris and 0% egg yolk), extender II (1.41% tris and 4% egg yolk), and extender III (2.41% tris and 8% egg yolk) to protect the semen progressive motility, total motility, viability, and functional integrity of membrane (by both HOST test). In addition, the semen progressive motility, total motility, and viability were not further increased, and the plasma membrane integrity (by both HOST tests) was decreased after supplementation of 4.41% tris and 24% egg yolk (extender V). This report suggests that the study of the presence of a lower amount of tris and egg yolk could not improve the frozen sperm quality than 3.41–4.41 gm tris and 16–24% egg yolk on semen extender for goats. We can minimize the tris and egg yolk concentration to 3.41% and 16%, respectively, to the previous studies, where they used 4.45 gm tris and 20% egg yolk-based extender for proper support of buck semen parameter after freezing and thawing [13,28]. 

A previous report showed that a pH between 7.2 and 8.2 provided higher SPM, STM, and SPMI than sperm with a pH between 5.2 and 6.2 [34]. The freezing of sperm resulted in an increase in ROS and oxidative stress-induced acidosis [51–53]. In addition, the pH of semen was decreased by the glycolytic pathway, which is the leading mechanism for energy production in semen, resulting in a decrease in metabolism and motility [8]. So, it is important to make the semen extender close to or slightly alkaline (like using tris) the environment of the semen of the body to overcome acidosis during freezing [19,54]. 

Furthermore, during the steps of cryopreservation and thawing, lipid peroxidation occurs due to ROS accumulation and the natural antioxidants’ removal from the seminal plasma, which could degrade the lipid tails of the plasma membrane of the sperm, resulting in undesirable effects on sperm functions [55]. The egg yolk and antioxidative enzymes in the extended semen defend against the loss of plasma membrane integrity [46], provide lipid [56], ion redistribution [57], intracellular enzyme release [58], and protect the semen from injury during the freeze-thawing process.

In addition, to minimize or overcome the acidosis [54,59,60] and egg yolk residue reaction with semen plasma, we recommended that, after washing, it is best to use 3.41% tris and 16% egg yolk in a fructose-ci­tric acid-glycerol-containing extender for buck semen cryopreservation.

Furthermore, the addition of tris (4.41 gm/100 ml) and egg yolk (24/100 ml) to the extender V did not significantly increase or decrease the result due to the harmful effect initiated by more tris, which caused toxicity and increased the outer membrane permeability of sperm by penetrating the sperm cell [61,62] and more egg yolk residues reaction with the remaining plasma during the freezing-thawing, described above.

The results of this investigation, however, disagree with the observations of Bispo et al. [15] and Shamsuddin et al. [63], which indicated that low egg yolk concentrations (2.5%) may be employed for buck sperm cryopreservation. This variation could result from using various goat breeds, seasons, and locations [41].

The result of this study may be attributed to a better balance among the different causes that impact the quality of semen throughout the different stages of freezing, improving the progressive motility, total motility, viability, and functional integrity of the plasma membrane.

## Conclusion

It may be concluded that after washing, the freezing extender supplemented with 3.41% tris and 16% egg yolk could be used for freezing buck sperm. In addition, this supplementation of extender enhanced the progressive motility, total motility, and viability of spermatozoa. Furthermore, the supplementation with 3.41% tris with 16% egg yolk also enhanced the functional integrity of sperm by using both the HOST (HOST under light microscopy and stained sperm after HOST under light microscopy). The study would be helpful for further strategies for cryopreservation and artificial insemination of buck spermatozoa.

## List of Abbreviations

BUSgp60, Glycoproteins-60; HOST, Hypoosmotic swelling test; LPC, Lysophosphatidylcholine; SPM, Sperm progressive motility; STM, Sperm total motility; SPMI, Sperm plasma membrane integrity; SV, Sperm viability; ROS, reactive oxygen species.

## References

[1] Januskauskas A, Johannisson A, Rodriguez-Martinez H (2003). Subtle membrane changes in cryopreserved bull semen in relation with sperm viability, chromatin structure, and field fertility. Theriogenology.

[2] Salamon S, Maxwell WMC (2000). Storage of ram semen. Anim Reprod Sci.

[3] Isachenko E, Isachenko V, Katkov II, Dessole S, Nawroth F (2003). Vitrification of mammalian spermatozoa in the absence of cryoprotectants: from past practical difficulties to present success. Reprod Biomed Online.

[4] Soltanpour F, Moghaddam G (2013). Effects of frozen diluents on storage of ram sperm. Int J Adv Biol Biom Res.

[5] Gangwar C, Saxena A, Patel A, Singh SP, Yadav S, Kumar R (2018). Effect of reduced glutathione supplementation on cryopreservation induced sperm cryoinjuries in Murrah bull semen. Anim Reprod Sci.

[6] Waberski D, Petrunkina AM, Busch W, Waberski D (2007). Spermatologie. Haus- nutztieren, taschenbuch – 1.

[7] Hahn K, Failing K, Wehrend A (2019). Effect of temperature and time after collection on buck sperm quality. BMC Vet Res.

[8] Rigau T, Piedrafita J, Reverter A, Canal M, Rodríguez-Gil JE (1996). The rate of L-lactate production: a feasible parameter for the fresh diluted boar semen quality analysis. Anim Reprod Sci.

[9] Gadea J (2003). Semen extenders used in the artificial inseminarion of swine. Span J Agric Res.

[10] Forouzanfar M, Sharafi M, Hosseini SM, Ostadhosseini S, Hajian M, Hosseini L (2010). In vitro comparison of egg yolk–based and soybean lecithin–based extenders for cryopreservation of ram semen. Theriogenology.

[11] Pellicer-Rubio MT, Combarnous Y (1998). Deterioration of goat spermatozoa in skimmed milk- based extenders as a result of oleic acid released by the bulbourethral lipase BUSgp60. J Reprod Fertil.

[12] Upreti GC, Hall EL, Koppens D, Oliver JE, Vishwanath R (1999). Studies on the measurement of phospholipase A2 (PLA2) and PLA2 inhibitor activities in ram semen. Anim Reprod Sci.

[13] Anand M, Baghel G, Yadav S (2017). Effect of egg yolk concentration and washing on sperm quality following cryopreservation in Barbari buck semen. J Appl Anim Res.

[14] Roca J, Carrizosa JA, Campos I, Lafuente A, Vazquez JM, Martinez E (1997). Viability and fertility of unwashed Murciano-Granadina goat spermatozoa diluted in Tris-egg yolk extender and stored at 5 C. Small Rumin Res.

[15] Bispo CAS, Pugliesi G, Galvão P, Rodrigues MT, Ker PG, Filgueiras B (2011). Effect of low and high egg yolk concentrations in the semen extender for goat semencryopreservation. Small Rumin Res.

[16] Naing SW, Haron AW, Goriman MAK, Yusoff R, Bakar MZA, Sarsaifi K (2011). Effect of seminal plasma removal, washing solutions, and centrifugation regimes on Boer goat semen cryopreservation. Pertanika J Trop Agri Sci.

[17] Ranjan R, Goel AK, Ramachandran N, Kharche SD, Jindal SK (2015). Effect of egg yolk levels and equilibration periods on freezability of Jamunapari buck semen. Indian J Small Ruminants.

[18] Ferreira VDS, Mello MRBD, Fonseca CEMD, Dias ÁCF, Cardoso JM, Silva RB (2014). Effect of seminal plasma and egg yolk concentration on freezability of goat semen. Revista Brasileira de Zootecnia.

[19] Namula Z, Tanihara F, Wittayarat M, Hirata M, Nguyen NT, Hirano T (2019). Effects of Tris (hydroxymethyl) aminomethane on the quality of frozen-thawed boar spermatozoa. Acta Vet Hung.

[20] Chauhan MS, Anand SR (1990). Effect of egg yolk lipids on the freezing of goat semen. Theriogenology.

[21] World Health Organization (2010). WHO laboratory manual for the examination and processing of human semen.

[22] Jeyendran RS, Van der Ven HH, Perez-Pelaez M, Crabo BG, Zaneveld LJD (1984). Development of an assay to assess the functional integrity of the human sperm membrane and its relationship to other semen characteristics. J Reprod Fertil.

[23] Revell SG, Mrode RA (1994). An osmotic resistance test for bovine semen. Anim Reprod Sci.

[24] Baiee FH, Wahid H, Rosnina Y, Ariff OM, Yimer N, Salman H (2017). Hypo-osmotic swelling test modification to enhance cell membrane integrity evaluation in cryopreserved bull semen. Pertanika J Trop Agric Sci.

[25] Yoshida T, Alam ME, Hanafusa K, Tsujimoto Y, Tsukamoto M, Kanegi R (2022). Effects of the preservation medium and storage duration of domestic cat ovaries on the maturational and developmental competence of oocytes in vitro. J Reprod Dev.

[26] Yousef A, Ghasemzadeh-Nava H, Tajik P, Akbarinejad V, Towhidi A (2022). Evaluation of soy lecithin efficacy in comparison with egg yolk on freezing of epididymal sperm in dogs. Iran J Vet Med.

[27] Hermansson U, Johannisson A, Axnér E (2021). Cryopreservation of dog semen in a Tris extender with two different 1% soybean preparations compared with a Tris egg yolk extender. Vet Med Sci.

[28] Sun L, Fan W, Wu C, Zhang S, Dai J, Zhang D (2020). Effect of substituting different concentrations of soybean lecithin and egg yolk in tris-based extender on goat semen cryopreservation. Cryobiology.

[29] Tsujimoto Y, Fujiki K, Alam ME, Tsukamoto M, Azuma R, Kanegi R (2019). Development of feline embryos produced by Piezo-actuated intracytoplasmic sperm injection of elongated spermatids. J Reprod Dev.

[30] De Leeuw FE, Chen HC, Colenbrander B, Verkleij AJ (1990). Cold-induced ultrastructural changes in bull and boar sperm plasma membranes. Cryobiology.

[31] Hammerstedt RH, Graham JK, Nolan JP (1990). Cryopreservation of mammalian sperm: what we ask them to survive. J Androl.

[32] Makarova NP, Romanov YA, Dolgushina NV, Parker MM, Krasnyi AM (2018). Comparative analysis of the expression of glutathione peroxidase and glutathione reductase genes in human sperm after cryopreservation. Bull Exp Biol Med.

[33] De Menezes EB, van Tilburg M, Plante G, de Oliveira RV, Moura AA, Manjunath P (2016). Milk proteins interact with goat binder of sperm (BSP) proteins and decrease their binding to sperm. Cell Tissue Res.

[34] Zhou J, Chen LI, Li J, Li H, Hong Z, Xie M (2015). The semen pH affects sperm motility and capacitation. PloS One.

[35] Bonato M, Cornwallis CK, Malecki IA, Rybnik-Trzaskowska PK, Cloete SW (2012). The effect of temperature and pH on the motility and viability of ostrich sperm. Anim Reprod Sci.

[36] Naz S, Umair M, Iqbal S (2019). Ostrich egg yolk improves post thaw quality and in vivo fertility of Nili Ravi buffalo (Bubalus bubalis) bull spermatozoa. Theriogenology.

[37] Mocé E, Blanch E, Tomás C, Graham JK (2010). Use of cholesterol in sperm cryopreservation: present moment and perspectives to future. Reprod Domest Anim.

[38] Bergeron A, Manjunath P (2006). New insights towards understanding the mechanisms of sperm protection by egg yolk and milk. Mol Reprod Dev.

[39] Moussa M, Martinet V, Trimeche A, Tainturier D, Anton M (2002). Low density lipoproteins extracted from hen egg yolk by an easy method: cryoprotective effect on frozen–thawed bull semen. Theriogenology.

[40] Lanz RN, Pickett BW, Komarek RJ (1965). Effect of lipid additives on pre-and post-freeze survival of bovine spermatozoa. J Dairy Sci.

[41] Leboeuf B, Restall B, Salamon S (2000). Production and storage of goat semen for artificial insemination. Anim Reprod Sci.

[42] Liu T, Han Y, Zhou T, Zhang R, Chen H, Chen S (2019). Mechanisms of ROS-induced mitochondria-dependent apoptosis underlying liquid storage of goat spermatozoa. Aging (Albany NY).

[43] Falchi L, Galleri G, Zedda MT, Pau S, Bogliolo L, Ariu F (2018). Liquid storage of ram semen for 96 h: effects on kinematic parameters, membranes and DNA integrity, and ROS production. Livest Sci.

[44] Desai NR, Mahfouz R, Sharma R, Gupta S, Agarwal A (2010). Reactive oxygen species levels are independent of sperm concentration, motility, and abstinence in a normal, healthy, proven fertile man: a longitudinal study. Fertil Steril.

[45] Müller K, Müller P, Pincemy G, Kurz A, Labbe C (2008). Characterization of sperm plasma membrane properties after cholesterol modification: consequences for cryopreservation of rainbow trout spermatozoa. Biol Reprod.

[46] Ortman K, Rodriguez‐Martinez H (1994). Membrane damage during dilution, cooling and freezing‐thawing of boar spermatozoa packaged in plastic bags. Zentralbl Veterinarmed A.

[47] Paulenz H, Söderquist L, Pérez-Pé R, Berg KA (2002). Effect of different extenders and storage temperatures on sperm viability of liquid ram semen. Theriogenology.

[48] Choe CY, Kim JG, Cho SR, Son DS, Kim YK, Balasubramanian S (2006). Influence of seasons, extenders, slow and rapid freezing on seminal characters in Korean native bucks. Reprod Domest Anim.

[49] Aboagla EME, Terada T (2004). Effects of egg yolk during the freezing step of cryopreservation on the viability of goat spermatozoa. Theriogenology.

[50] Ritar AJ, Salamon S (1982). Effects of seminal plasma and of its removal and of egg yolk in the diluent on the survival of fresh and frozen-thawed spermatozoa of the Angora goat. Aust J Biol Sci.

[51] Lucio CF, Regazzi FM, Silva LCG, Angrimani DSR, Nichi M, Vannucchi CI (2016). Oxidative stress at different stages of two-step semen cryopreservation procedures in dogs. Theriogenology.

[52] Schieber M, Chandel NS (2014). ROS function in redox signaling and oxidative stress. Curr Biol.

[53] Tsai KL, Wang SM, Chen CC, Fong TH, Wu ML (1997). Mechanism of oxidative stress‐induced intracellular acidosis in rat cerebellar astrocytes and C6 glioma cells. J Physiol.

[54] Giunti C, Priouzeau F, Allemand D, Levraut J (2007). Effect of Tris-hydroxymethyl aminomethane on intracellular pH depends on the extracellular non-bicarbonate buffering capacity. Transl Res.

[55] Bansal AK, Bilaspuri GS (2011). Impacts of oxidative stress and antioxidants on semen functions. Vet Med Int.

[56] Darin-Bennett A, Poulos A, White IG (1973). The effect of cold shock and freeze-thawing on release of phospholipids by ram, bull, and boar spermatozoa. Aust J Biol Sci.

[57] Quinn PJ, White IG (1968). The effect of pH, cations and protective agents on the susceptibility of ram spermatozoa to cold shock. Exp Cell Res.

[58] Harrison RAP, White IG (1972). Glycolytic enzymes in the spermatozoa and cytoplasmic droplets of bull, boar and ram, and their leakage after shock. J Reprod Fertil.

[59] Ugur MR, Saber Abdelrahman A, Evans HC, Gilmore AA, Hitit M, Arifiantini RI (2019). Advances in cryopreservation of bull sperm. Front Vet Sci.

[60] Baust JG, Gao D, Baust JM (2009). Cryopreservation: an emerging paradigm change. Organogenesis.

[61] Good NE, Winget GD, Winter W, Connolly TN, Izawa S, Singh RM (1966). Hydrogen ion buffers for biological research. Biochemistry.

[62] Irvin RT, MacAlister TJ, Costerton JW (1981). Tris (hydroxymethyl) aminomethane buffer modification of *Escherichia coli* outer membrane permeability. J Bacteriol.

[63] Shamsuddin M, Amiri Y, Bhuiyan MMU (2000). Characteristics of buck semen with regard to ejaculate numbers, collection intervals, diluents and preservation periods. Reprod Domest Anim.

